# Nervanin

**Published:** 1899-11

**Authors:** A. D. Kyner

**Affiliations:** Blue Mound, Ill.


ARTICLE IV.

### NERVANIN.

DR. A. D. KYNER, BLUE MOUND, ILL.

Within the past year orthoform has filled a place that has, heretofore, been practically unoccupied by any remedy possessing the necessary qualifications of a typical anæsthetic. Such a remedy should be non-poisonous and of slow solubility. None in use was non-toxic; all were too rapidly absorbed and their action of but short duration. The slow absorption of orthoform by the tissues, its absolute non-toxity and antiseptic properties fulfill all the requirements for such a preparation. The great success attending its use made it most desirable to obtain this salt in a soluble form for subcutaneous injections. The inventors, Prof. Hinborn and Dr. Heinz, have succeeded in obtaining a soluble compound of neutral reaction, and have named it "Nervanin." Experiments have demonstrated that nervanin is but one-tenth as toxic as cocaine. Eight grains was found to be the maximum dose that could be administered subcutaneously without injury, and the experience of Drs. W. Rotenberger, Marcus and others confirm its practical non toxicity.

For the extraction of teeth, a 5 per cent. solution is employed, and like eucaine, the solution may be boiled without decomposition. A sterilizing agent need not be added, as nervanin in 1 per cent. solution prevents bacterial growth.

I believe that a 5 per cent. solution of nervanin, if properly injected into ordinarily healthy gum tissue and allowed to act for from three to five minutes before operating, will produce a deeper, more profound and more prolonged anæsthesia than either cocaine or eucaine B. In extracting difficult roots, patients have experienced no pain when the alveolar process was cut through to grasp the root. I have never obtained this immunity from pain with other anæsthetics, and therefore believe that it penetrates deeply. The anæsthetic effect is prolonged for a number of hours after operating, as careful inquiries made of patients for whom I had extracted record absolute freedom from after pain. Careful attention was given to the gums to note whether any irritation was produced, and in every case examined the gums rapidly returned to a normal condition without œdema or sloughing. The non-irritating properties thus shown will especially recommend themselves to all who do not wish to treat "sore gums."—*Items of Interest.*

